# 

**Published:** 1880-07

**Authors:** 


### CONTENTS.

COMMUNICATIONS.

Dental Education ........ 293
What Do You Do it For ........ 299
Address ........ 304
Pathology and Semiology ........ 310
Cylinder Filling ........ 316
Tardy Eruption of the Teeth ........ 318
Clinic Reports ........ 320
Scientific News ........ 321
Correspondence ........ 325

EDITORIAL.

An Important Movement ........ 327
Call for a Mass Convention ........ 328
Dennett's Dental Naboli ........ 331
A New Appliance ........ 335
Bibliographical ........ [illegible]36
Obituary ........ 336
Excursion ........ 337

